# Anti-inflammatory effects of *Banisteriopsis caapi* and beta-carbolines in neuronal cells: potential implications for neuro-COVID

**DOI:** 10.3389/fphar.2025.1703727

**Published:** 2026-01-26

**Authors:** Laura Seixas Bianco, Tiago Nicoliche, Nelson Francisco Corrêa-Neto, Rafael Lanaro, Ariadiny L. Caetano, Carla Maximo Prado, Rodrigo Portes Ureshino, Iolanda de Fátima Lopes Calvo Tibério, Renato Fraga Righetti, Roberta Sessa Stilhano, Alessandra Linardi

**Affiliations:** 1 Department of Physiological Sciences, Santa Casa de São Paulo School of Medical Sciences, São Paulo, Brazil; 2 Poison Control Center, Faculty of Medical Sciences, State University of Campinas (UNICAMP), Campinas, Brazil; 3 Department of Bioscience, Federal University of São Paulo (UNIFESP), Santos, Brazil; 4 Department of Biological Sciences, Federal University of São Paulo (UNIFESP), Diadema, Brazil; 5 Laboratory of Molecular and Translational Endocrinology, Escola Paulista de Medicina, Federal University of São Paulo (UNIFESP), São Paulo, SP, Brazil; 6 Faculty of Medicine, University of São Paulo (USP), São Paulo, Brazil; 7 Rehabilitation Service, Hospital Sírio-Libanês, São Paulo, Brazil

**Keywords:** *Banisteriopsis caapi*, beta-carbolines, harmaline, harmine, neuroinflammation, SARS-CoV-2

## Abstract

**Background:**

Neuroinflammation plays a central role in neurodegenerative diseases such as Alzheimer’s and Parkinson’s, along with depression, anxiety, and infectious diseases including COVID-19. Harmine and harmaline, β-carboline alkaloids from *Banisteriopsis caapi*, exhibit immunomodulatory, anti-inflammatory, and neuroprotective properties. In this study, we aimed not only to investigate the anti-inflammatory and neuroprotective effects of β-carbolines and *B. caapi* extract on a lipopolysaccharide (LPS)-induced neuroinflammation model using SH-SY5Y cells and their impact on severe acute respiratory syndrome coronavirus 2 (SARS-CoV-2) receptor expression but also to compare cytokine levels in plasma from intensive care unit (ICU) and non-ICU COVID-19 patients, thereby providing clinical context for the inflammatory response.

**Methods:**

SH-SY5Y cells were treated with LPS and incubated with harmine, harmaline, or *B. caapi* extract. Cell viability was assessed using the MTT assay. Cytokine expression was quantified by ELISA, and receptor gene expression was analyzed using RT-qPCR. Plasma was obtained from the Hospital das Clínicas of the University of São Paulo Medical School (HCFMUSP) biobank.

**Results:**

IL-6 was high in ICU patients; LPS increased IL-6 and TNF-α cytokine levels in cells, whereas harmine and harmaline significantly reduced both cytokines. *B. caapi* extract decreased LPS-induced NF-κB and TNF-α but did not affect IL-6. Harmine also reduced NF-κB expression. None of the treatments altered TMPRSS11D or furin, and harmaline showed no effect on ACE2. In contrast, the extract upregulated ACE2, whereas harmine induced a modest increase. Only harmine and harmaline reduced TMPRSS2 expression.

**Conclusion:**

β-carbolines and *B. caapi* extract attenuate LPS-induced cytokine production in SH-SY5Y cells, supporting their anti-inflammatory and neuroprotective potential. The extract and β-carbolines also modulate ACE2 and TMPRSS2 expressions, suggesting relevance to mechanisms associated with neuro-COVID.

## 1 Introduction

Neuroinflammation is a key factor in the development and progression of neurodegenerative diseases, such as Alzheimer’s disease, Parkinson’s disease, and multiple sclerosis (Glass et al., 2010). When improperly activated, the inflammatory response can lead to acute or chronic diseases. When it occurs in the central nervous system (CNS), it may result in the development of neuropsychiatric conditions such as depression, anxiety, cognitive disorders, and neurodegenerative diseases (Miller et al., 2009; Glass et al., 2010; Benros et al., 2013). Under some conditions, neuroinflammation is a key component and also a risk factor of the neurophysiopathological process (Benros et al., 2013; da Silva et al., 2021). Furthermore, the literature has shown that neuroinflammation can be observed in infectious diseases, such as in corona virus disease (COVID)-19 (Cantini et al., 2020; Huang et al., 2020; Mehta et al., 2020; Mulchandani et al., 2021; Zhang et al., 2021).

Severe acute respiratory syndrome coronavirus 2 (SARS-CoV-2) is the etiological agent responsible for COVID-19, an illness that primarily affects the respiratory system and ranges from mild symptoms to severe respiratory failure (Lamers and Haagmans, 2022). SARS-CoV-2 can infect cells through various mechanisms. One example is the interaction between angiotensin-converting enzyme 2 (ACE2) and the virus’s spike (S) protein, facilitating its attachment (Hörnich et al., 2021). Moreover, transmembrane serine protease II (TMPRSS2) leads to membrane fusion in both the virus and the cell (Fraser et al., 2022). Another mechanism involves the cleavage of S protein by TMPRSS2, TMPRSS11D, or the cellular protease furin into two subunits, S1 and S2. After binding to ACE2, S1 remains attached to ACE2, whereas S2 is cleaved into S2′, thus promoting the fusion of the viral envelope with the host cell membrane (Glowacka et al., 2011; Hoffmann et al., 2020; Kishimoto et al., 2021). Furthermore, high levels of pro-inflammatory cytokines, such as TNF-α (Huang et al., 2020), have been detected in COVID-19 patients, and the resulting cytokine storm in the lungs may lead to patient mortality (Mehta et al., 2020). Therefore, inflammation is one of the most important symptoms of COVID-19, and the use of drugs that modulate inflammation is clinically relevant. Studies on the inflammatory response in COVID-19 have found higher levels of IL-2 (Cantini et al., 2020), IL-4, IL-5 (Zhang et al., 2021), IL-6 (Cantini et al., 2020), IL-8, IL-10, IL-2R, TNF-α (Mulchandani et al., 2021), and IFN-γ (Zhang et al., 2021). Additionally, literature data also show that SARS-CoV-2 is capable of infecting the CNS and peripheral nervous system (PNS) (Natoli et al., 2020; Brann et al., 2020; Paniz-Mondolfi et al., 2020), inducing neurological symptoms such as dizziness, headaches, nausea, vomiting, hypogeusia, hypopsia, and hyposmia. Thus, oxidative stress and neuroinflammation have emerged as potential contributors to the neurological symptoms observed in COVID-19.

*Banisteriopsis caapi* (family: Malpighiaceae) is a tropical South American liana, found mainly in Brazil, Bolivia, Colombia, Ecuador, and Peru, which is associated with *Psychotria viridis* to prepare the psychoactive beverage Ayahuasca (McKenna and Riba, 2015). The therapeutic potential of the *B. caapi* extract has been demonstrated in diseases such as Parkinson’s disease and depression (Samoylenko et al., 2010). The primary alkaloids found in *B. caapi* are β-carbolines harmine, harmaline, and tetrahydroharmine (Callaway et al., 1996; Yritia et al., 2002; McKenna and Riba, 2015). Fortunato et al. (2009) reported an antidepressant effect in rats following acute administration of harmine. They also observed an increase in brain-derived neurotrophic factor (BDNF) levels in the hippocampus of rats after harmine administration. Harmine additionally reversed adrenal gland hypertrophy and anhedonia while normalizing adrenocorticotropic hormone (ACTH) levels (Fortunato et al., 2010). Moreover, β-carbolines are derived from tryptophan and, as a result, can also exhibit antioxidant, anxiogenic, or anxiolytic effects depending on the dose (Tse et al., 1991; McKenna, 2004; Farzin and Mansouri, 2006). It has been reported that β-carbolines can also act on GABA_A_ receptors for gamma-aminobutyric acid (GABA) as inverse agonists, and it has also been shown that β-carbolines influence 5-HT_2A_ and 5-HT_2C_ receptors, increasing dopamine efflux (Venault and Chapouthier, 2007). In addition, harmine, harmaline, and tetrahydroharmine have been demonstrated to promote neurogenesis in neurospheres prepared from progenitor cells harvested from the subventricular zone and dentate gyrus of adult mice (Morales-García et al., 2017). All β-carbolines stimulated proliferation, migration, and differentiation of neural stem cells into adult neurons. Additionally, harmine can inhibit the activation of NF-κB induced by lipopolysaccharide (LPS) and TNF-α while reducing protein levels of IL-1β, TNF-α, and IL-6 both *in vivo* (in mice) and *in vitro* (in RAW 264.7 macrophage cells) (Xin et al., 2017). Jin et al. (2022) also demonstrated that harmine inhibited inducible NOS, COX-2, TNF-α, IL-6, IL-12, and other markers in LPS-induced BALB/c and C57BL/6 mouse macrophages. The hypothesis that harmaline and its analogs may inhibit COX-2 and thereby exert anti-inflammatory effects has also been explored. Uddin et al. (2020) designed, synthesized, and tested several harmaline analogs, observing that they inhibited purified human COX-2.

The SH-SY5Y neuroblastoma cell line has become a popular cell model for neurological disease studies. The SH-SY5Y cell line exhibits neuronal marker enzyme activities (such as tyrosine hydroxylase and dopamine-β-hydroxylase), specific uptake of norepinephrine, and expression of one or more neurofilament proteins, as well as opioid receptors, muscarinic receptors, and nerve growth factor receptors (Xie et al., 2010). Furthermore, SH-SY5Y cells are able to proliferate in culture for extended periods without contamination (Xie et al., 2010). Because of these properties, SH-SY5Y cells are extensively used in neurological research, including investigations into neuronal differentiation, metabolism, neurodegenerative processes, neuroadaptive responses, neurotoxicity, and neuroprotection (Xie et al., 2010; Ma et al., 2019). In addition, these cells have been used in the literature as a model for neuroinflammation. Thus, studies have described LPS incubation for inducing neuroinflammation in SH-SY5Y cells (Park et al., 2019; Saisawang et al., 2023). Endotoxin or LPS is the major component of the outer membrane of bacterial cells that triggers the activation of pro-inflammatory cytokines (Lu et al., 2008). Consequently, the incubation of neuronal cells with LPS can be a good *in vitro* neuroinflammation model.

Therefore, considering the central role of the inflammatory response in COVID-19 pathophysiology and its impact on neurological outcomes, along with the previously reported anti-inflammatory and neuroprotective properties of β-carbolines and *B. caapi* extract (Moloudizargari et al., 2013; Mulchandani et al., 2021), we hypothesized that these compounds could attenuate neuroinflammation associated with the disease. Thus, in this study, we aimed to (i) evaluate the anti-inflammatory effects of *B. caapi* extract, harmine, and harmaline on LPS-induced cytokine production in SH-SY5Y neuroblastoma cells, (ii) investigate their modulatory effects on key SARS-CoV-2 entry receptors (ACE2, TMPRSS2, TMPRSS11D, and furin), and (iii) compare plasma levels of the inflammatory cytokine IL-6 in intensive care unit (ICU) and non-ICU COVID-19 patients to provide clinical context on the relevance of IL-6 in COVID-19 severity, while recognizing that these observations are not intended as direct validation of the *in vitro* experiments.

## 2 Materials and methods

### 2.1 *Banisteriopsis caapi* extract preparation and quantification of β-carbolines

The *B. caapi* (Spruce ex Griseb.) Morton extract (https://worldfloraonline.org) was provided by the Centro Integrado Beneficente Caminho do Vegetal (CIBCV) (Monte Mor, SP, Brazil) (Sisgen registration A2FCD8D). To prepare the extract, liana *B. caapi* was carefully washed in water and pounded with wooden mallets. The plant material (1 kg) was boiled (three times) and concentrated over several hours to produce approximately 2 L of beverage. The harmine and harmaline standards were obtained from Sigma-Aldrich (Steinheim, Germany). Tetrahydroharmine was provided by Dr. Mauricio Yonamine (FCF, USP). Stock solutions (1.0 mg/mL) of harmine, harmaline, and tetrahydroharmine were prepared with methanol and stored in a freezer at −20 °C. The quantification of β-carbolines in the extract sample was performed using HPLC-DAD (Shimadzu Prominence model), with a quaternary pump, automatic sampler, and an Atlantis T3 150 × 3.0 mm × 3 µm column (Waters), thermostated at 35 °C (Lanaro et al., 2015). In summary, 200 μL of each sample was vigorously mixed with 800 μL of methanol using a vortex and centrifuged. An aliquot of 200 μL of the supernatant was transferred to autosampler glass vials containing 800 μL of methanol. Chromatographic separation was performed using a mobile phase gradient consisting of (A) 10 mmol/L phosphoric acid in ultrapure water (pH 3.0) and (B) acetonitrile, delivered at a flow rate of 1 mL/min. The gradient started at 40% A and 60% B for 1 min, followed by a linear shift to 5% A and 95% B over 13 min. For quantification, the chromatograms were extracted at 291 nm (THH), 320 nm (harmine), and 375 nm (harmaline). The method was linear from 1 to 100 μg/mL for all three alkaloids. The intraday and interday precision and accuracy showed relative standard deviations (RSDs) of <10% for all QCs. The limits of detection (LOD) and quantification (LOQ) for all analytes were determined as 0.5 μg/mL and 1.0 μg/mL, respectively. The LOD and LOQ were determined by analyzing decreasing concentrations until signal-to-noise (S/N) ratios >3 (LOD) and >10 (LOQ) were obtained for all analytes. Table 1 shows the details of the characterization of the *B. caapi* extract.

**TABLE 1:** Concentrations of β-carbolines (harmine, harmaline, and THH) in the *B. caapi* extract.

Mean concentration (µg/mL, n = 3)			
​	Harmine	Harmaline	THH
*B. caapi* extract	650.0	126.0	380.0

THH, tetrahydroharmine; LOD, limit of detection (0.5 μg/mL); LOQ, limit of quantification (1.0 μg/mL).

### 2.2 Cytokine quantification in plasma samples

The plasma was obtained from an existing sample bank at Hospital das Clínicas of the University of São Paulo Medical School (HCFMUSP), São Paulo, Brazil (approved by the research ethics committee CEP 4.423.008). The volunteers were men and women hospitalized in the ICUs and wards of the HCFMUSP, and had been diagnosed with COVID-19, from March 2020 to October 2020. The patients were classified as critical and non-critical, with 21 men and 21 women in each group, totaling 42 volunteers per group. The volunteers had previously signed a free and informed consent form to participate in research projects. IL-6 was analyzed in plasma of ICU and non-ICU patients using an ELISA kit (R&D) according to the manufacturer’s instructions.

### 2.3 Cell culture

The SH-SY5Y neuroblastoma cell line was cultured in Dulbecco’s modified Eagle’s medium (DMEM, Gibco, MA, United States) with high glucose, supplemented with 10% fetal bovine serum (FBS), 100 U/mL penicillin, and 100 U/mL streptomycin, hereafter referred to as supplemented DMEM (sDMEM). Cells were maintained at 37 °C in a humidified incubator with 5% CO_2_ (Hasegawa et al., 2004).

### 2.4 Cell viability assay

Cell viability was determined using a colorimetric assay based on 3-(4,5-dimethylthiazol-2-yl)-2,5-diphenyl tetrazolium bromide (MTT; Sigma-Aldrich, United States). SH-SY5Y cells were seeded in 24-well plates at a density of 1.0 × 10^5^ cells/well and incubated with 500 µL of sDMEM containing 5 mg/mL MTT at 37 °C for 3 h. The resulting formazan crystals were solubilized in 500 µL of dimethyl sulfoxide (DMSO), and optical density was measured at 590 nm using a SpectraMax M microplate reader (Molecular Devices®).

### 2.5 Treatment with *B. caapi* extract, harmine, and harmaline following LPS-induced inflammation

Neuroinflammation was induced by treating SH-SY5Y cells with lipopolysaccharide (Sigma-Aldrich, United States) for 24 h or 48 h. The half-maximal inhibitory concentration (IC_50_) of LPS was determined to be 175 μg/mL at 24 h and 130 μg/mL at 48 h. SH-SY5Y cells were seeded in 24-well plates at a density of 5.0 × 10^4^ cells per well. Culture supernatants were collected after 24 h and 48 h of LPS exposure. Based on the IC_50_ values, the 48-h time point and the concentration of 130 μg/mL were selected for subsequent experiments.

Regarding treatment with *B. caapi* extract and β-carbolines, SH-SY5Y cells were seeded in 12-well plates at a density of 2.0 × 10^5^ cells/well. After 24 h, cells were pretreated with harmine or harmaline (3 or 10 µM) or *B. caapi* extract (0.1%) for an additional 24 h. Subsequently, LPS (130 μg/mL) was added to the culture medium, and cells were incubated for another 48 h, totaling 72 h of treatment. Following incubation, culture supernatants were collected for cytokine quantification, and cells were harvested and stored for RT-qPCR analysis.

### 2.6 Quantification of inflammatory cytokines by ELISA

The levels of IL-6 and TNF-α were measured in the cell supernatant collected after treatment with *B. caapi* extract, harmine, and harmaline following LPS-induced inflammation, as previously described. The analysis was performed using an ELISA kit (R&D), following the manufacturer’s instructions.

### 2.7 Gene expression evaluation by RT-qPCR

RNA was extracted from the cells using TRIzol reagent (Thermo Fisher, MA, United States), and cDNA was synthesized using the high-capacity kit (Thermo Fisher), following the manufacturer’s instructions. Once the cDNA was obtained, the RT-qPCR reaction was performed with SYBR-Green (Qiagen, Hilden, Germany) following the manufacturer’s instructions. The primers used were as follows: NF-ĸB (NF-ĸB_F: 5′-TGG GAA GGC CTG AAC AAA TG-3′ and NF-ĸB_R: 5′-AGT GCC ATC TGT GGT TGA AA-3′), ACE2 (ACE2_F: 5′-TCC ATT GGT CTT CTG TCA CCC G-3′ and ACE_R: 5′-AGA CCA TCC ACC TCC ACT TCT C-3′), TMPRSS2 (TMPRSS2_F: 5′-CCT CTA ACT GGT GTG ATG GCG T-3′ and TMPRSS2_R: 5′-TGC CAG GAC TTC CTC TGA GAT G-3′), TMPRSS11D (TMPRSS11D_F: 5′-GGA GCC ATC TTG TCT GGA ATG C-3′ and TMPRSS11D_R: 5′- AAC CAA AGC CGC CGT GAG TCT T-3′), furin (Furin_F: 5′-GCC ACA TGA CTA CTC CGC AGA T-3′ and Furin_R: 5′-TAC GAG GGT GAA CTT GGT CAG C-3′), and R18Sh (R18Sh_F: 5′-ACC CGT TGA ACC CCC ATT CGT GA-3′ and R18Sh_R: 5′-GCC TCA CTA AAC ATC CAA TCG G-3′).

### 2.8 Statistical analysis

The results were expressed as mean ± SD (standard deviation). Statistical analysis was performed using GraphPad Prism version 8.0 (GraphPad Software, Inc., La Jolla, CA, United States). Data were analyzed using two-way ANOVA followed by Sidak’s *post hoc* test or one-way ANOVA followed by Tukey’s *post hoc* test or followed by Dunnett’s *post hoc* test. The Mann–Whitney test was also used to analyze volunteer’s data. Results were considered statistically significant if p < 0.05. The IC50 of LPS was estimated using the log-logistic regression three-parameter model through the *drc* (dose–response curve) R package (Ritz et al., 2015). This model elucidates the associations between various doses of a given compound and their respective biological responses, facilitating the determination of the IC50. R software, version 4.4.0 (R Foundation, Vienna, Austria), was used for this analysis, and a significance level of 5% was adopted.

## 3 Results

### 3.1 Differential expression of IL-6 in COVID-19 severity groups

**FIGURE 1:**
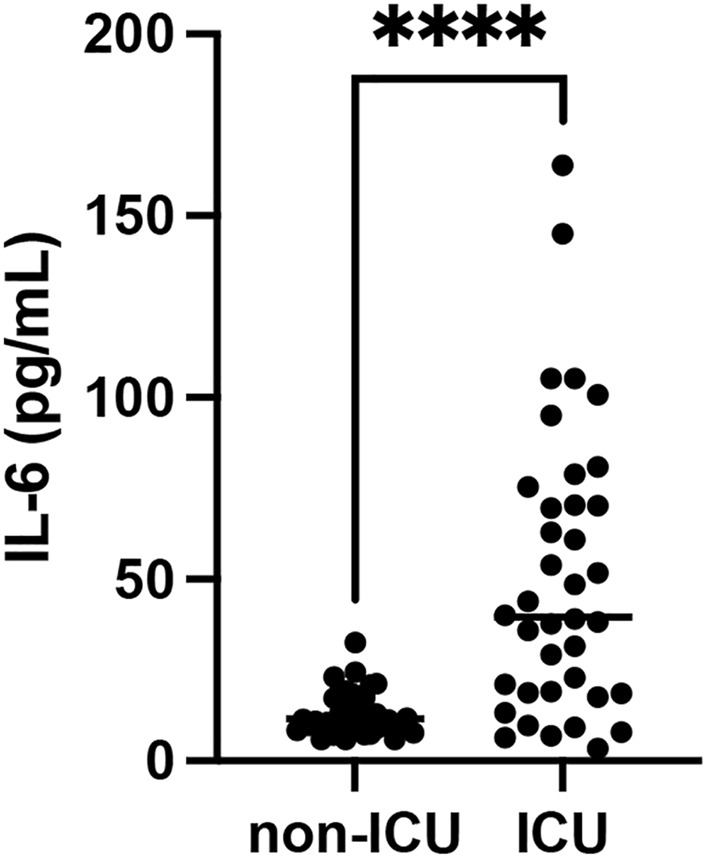
Quantification of IL-6 levels in plasma samples from ICU and non-ICU COVID-19 patients. Cytokine concentrations were determined by ELISA. IL-6 levels. Data are shown as scatter dot plots; each point represents an individual patient (n = 32–39). ****p < 0.0001, comparison between ICU and non-ICU groups.

Among the various cytokines involved in the inflammatory response to viral infections, IL-6 plays central roles in the pathophysiology of COVID-19 and is closely associated with disease severity and clinical outcomes. Therefore, this cytokine was selected for analysis in patient plasma to evaluate their association with disease severity. Plasma samples obtained from a biobank of previously hospitalized patients revealed detectable levels of IL-6 in both ICU and non-ICU groups. IL-6 concentrations were significantly higher in ICU patients (50.24 ± 38.94 pg/mL) than in non-ICU patients (13.43 ± 6.00 pg/mL), as shown in Figure 1.

### 3.2 Effect of *B. caapi* extract, β-carbolines, and LPS on SH-SY5Y cell viability

**FIGURE 2:**
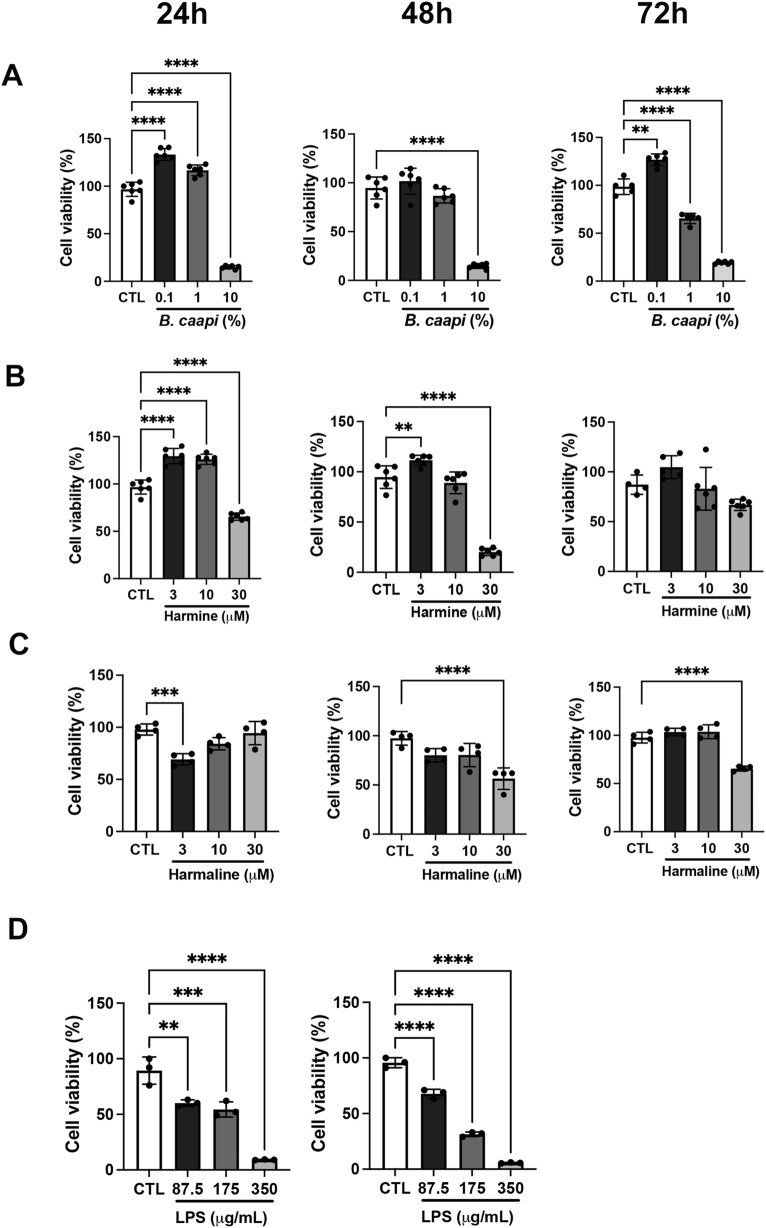
Viability of SH-SY5Y cells following treatment with *B. caapi* extract, harmine, harmaline, or LPS. Cell viability (%) was measured via the MTT assay and normalized to the untreated control (CTL = 100%). Treatments: **(A)** *B. caapi* extract; **(B)** harmine; **(C)** harmaline; **(D)** lipopolysaccharide (LPS). Data are expressed as mean ± SD (n = 3–6). Statistical significance: **p < 0.01, ***p < 0.001, and ****p < 0.0001.

Given that IL-6 levels were differentially modulated in ICU and non-ICU patients and motivated by the clinical importance of IL-6 in severe cases, we sought to investigate whether *B. caapi* extract and β-carbolines exert immunomodulatory effects *in vitro* in neuronal cells. As a first step, we assessed the cytotoxicity of these compounds in SH-SY5Y cells to determine appropriate nontoxic concentrations for subsequent experiments. Cell viability was evaluated using the MTT assay. The *B. caapi* extract did not affect cell viability at 0.1% at any time point; however, significant reductions in viability were observed at 1% and 10% compared to the untreated control (set as 100% viability) (Figure 2A). Harmine was nontoxic at concentrations ranging from 3 to 30 µM after 72 h, with 3 µM significantly increasing cell viability relative to the control (Figure 2B). However, the 30 µM concentration induced toxicity at 24 and 48 h. Harmaline showed no cytotoxicity at 3 and 10 μM, but it significantly decreased viability at 30 µM (Figure 2C). As expected, LPS treatment significantly reduced cell viability at all tested concentrations, consistent with its known cytotoxic and pro-inflammatory effects on neuronal cells (Figure 2D). To quantify its cytotoxic potency, IC_50_ was calculated. The IC_50_ values for LPS were 175.5 μg/mL (104–247.1, p < 0.001) at 24 h and 128.4 μg/mL (118.2–138.6, p < 0.001) at 48 h, indicating a time-dependent increase in cytotoxicity.

### 3.3 LPS successfully induces neuroinflammation in SH-SY5Y cells

**FIGURE 3:**
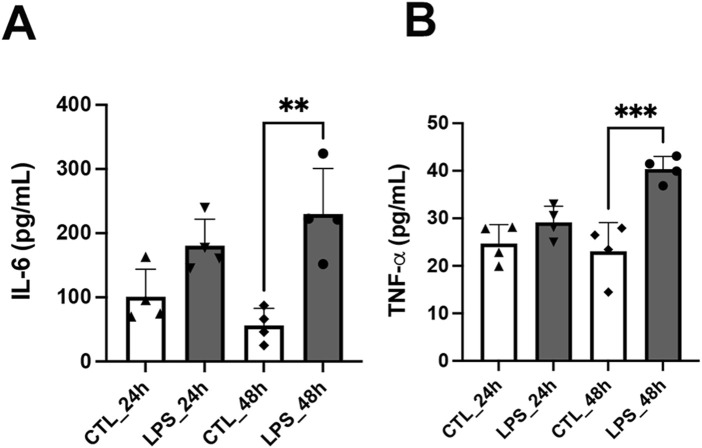
LPS-induced cytokine production in SH-SY5Y cells as a model of neuroinflammation. SH-SY5Y cells were treated with LPS at IC_50_ concentrations (175 μg/mL for 24 h and 130 μg/mL for 48 h), and cytokine levels were measured by ELISA. **(A)** IL-6 levels in the control and LPS-treated cells. **(B)** TNF-α levels in the control and LPS-treated cells. Data are expressed as mean ± SD (n = 4). **p < 0.01 and ***p < 0.001.

Following the determination of the IC_50_ for LPS (175 μg/mL at 24 h and 130 μg/mL at 48 h), these concentrations were used to induce neuroinflammation in SH-SY5Y cells. An ELISA assay was subsequently conducted to quantify IL-6, and TNF-α levels were also assessed at both time points. No significant alterations in cytokine levels were observed 24 h following LPS treatment. However, after 48 h, IL-6 levels were significantly elevated in the LPS group (229.9 ± 70.96 pg/mL) compared to the control group (56.22 ± 26.57 pg/mL) (Figure 3A). Similarly, TNF-α levels increased from 23.08 ± 6.00 pg/mL in the control group to 40.34 ± 2.66 pg/mL in the LPS-treated group (Figure 3B).

### 3.4 *B. caapi* extract, harmine, and harmaline attenuate LPS-induced neuroinflammation in SH-SY5Y cells

**FIGURE 4:**
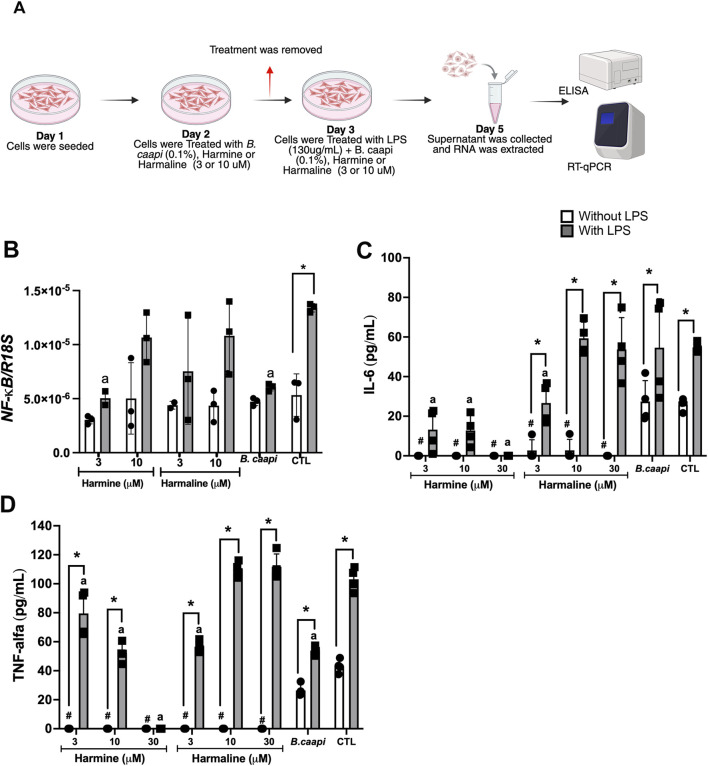
Anti-inflammatory effects of *B. caapi* extract, harmine, and harmaline on LPS-induced cytokine production in SH-SY5Y cells. SH-SY5Y cells were pretreated for 24 h with harmine or harmaline (3–10 µM) or *B. caapi* extract (0.1%), followed by stimulation with LPS (130 μg/mL) for 48 h to induce neuroinflammation. Cytokine levels were measured in the culture supernatant using ELISA. **(A)** Schematic representation of the treatment. **(B)** NF-κB expression. **(C)** IL-6 levels. **(D)** TNF-α levels. Results are presented as mean ± standard deviation (n = 3–4). *p < 0.05 vs. control without LPS for the same treatment; #p < 0.05 vs. control without LPS; a p < 0.05 vs. LPS-treated control.

To investigate the potential immunomodulatory effects of harmine, harmaline, and *B. caapi* extract, we selected the 48-h time point to assess their impact on NF-κB, IL-6, and TNF-α production in SH-SY5Y cells stimulated with LPS (130 μg/mL). The experimental design is illustrated in Figure 4A. The *B. caapi* extract (0.1%) and harmine (3 µM) modulated the NF-κB pathway at the transcriptional level. LPS stimulation significantly increased NF-κB mRNA expression, whereas incubation with *B. caapi* extract and harmine markedly reduced this effect (Figure 4B). Additionally, harmine and harmaline (3 and 10 μM) significantly reduced the basal IL-6 concentration (25.9 pg/mL) to undetectable levels (Figure 4C). With respect to the LPS-induced IL-6 concentration (54.7 pg/mL), harmine markedly reduced IL-6 levels to 13.2 pg/mL and 12.8 pg/mL at concentrations of 3 µM and 10 μM, respectively (Figure 4B). Basal IL-6 levels were reduced to 2.7 pg/mL following treatment with harmaline at both 3 µM and 10 μM concentrations (Figure 4C). Additionally, harmaline at 3 μM decreased the LPS-induced IL-6 concentration to 26.7 pg/mL. Harmine, at concentrations of 3 µM and 10 μM, markedly suppressed basal TNF-α levels (43.1 pg/mL) to nearly undetectable values (Figure 4D). In addition, harmine significantly reduced the LPS-induced TNF-α level (103 pg/mL) to 79.5 pg/mL and 52.2 pg/mL at 3 μM and 10 μM concentrations, respectively (Figure 4D). Additionally, harmaline (both concentrations) reduced the basal TNF-α level to zero and the LPS-induced TNF-α level to 56.6 pg/mL at 3 μM concentration (Figure 4D). The *B. caapi* extract, at a concentration of 0.1%, reduced TNF-α induced by LPS to 53.9 pg/mL, but there was no difference in the basal release of TNF-α (Figure 4D). Regarding IL-6, there was no change in basal and LPS-induced levels after incubation with 0.1% of the *B. caapi* extract (Figure 4C). These findings suggest that the extract and β-carbolines can interfere with NF-κB-dependent transcription and consequently reduce downstream cytokine production.

### 3.5 Differential modulation of SARS-CoV-2 entry receptors by harmine, harmaline, and *B. caapi* extract in SH-SY5Y cells

**FIGURE 5:**
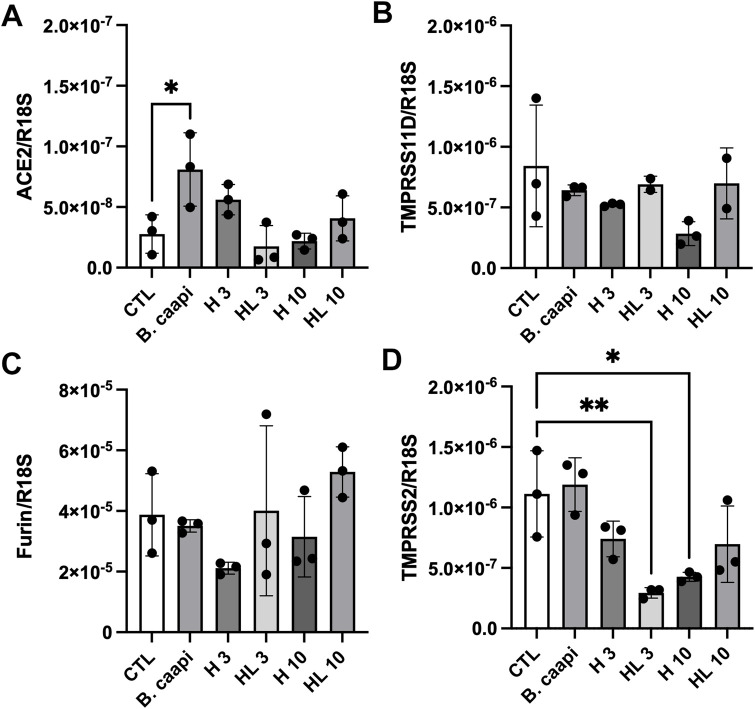
Expression of ACE2 **(A)**, TMPRSS11D **(B)**, furin **(C)**, and TMPRSS2 **(D)** receptors in SH-SY5Y cells treated with harmine, harmaline, and *B. caapi* extract followed by LPS-induced neuroinflammation. SH-SY5Y cells were pretreated with harmine or harmaline (10 µM) and *B. caapi* extract (0.1%) for 24 h, followed by incubation with LPS (130 μg/mL) for 48 h. Gene expression was assessed by RT-qPCR using total RNA extracted at the end of the LPS exposure. Results are expressed as mean ± standard deviation (n = 3–4). CTL: control group without treatment. *p < 0.05 vs. CTL; **p < 0.01 vs. CTL.

To explore a potential antiviral mechanism of action, we evaluated the expression of key viral entry receptors in SH-SY5Y cells treated with nontoxic concentrations of harmine and harmaline (3 and 10 µM), along with the *B. caapi* extract (0.1%). The mRNA expression levels of ACE2 (Figure 5A), TMPRSS11D (Figure 5B), furin (Figure 5C), and TMPRSS2 (Figure 5D) were analyzed using RT-qPCR. Harmine, harmaline, and the *B. caapi* extract did not significantly affect the expression of TMPRSS11D or furin. Under the same experimental conditions, harmaline also failed to alter ACE2 expression. In contrast, the *B. caapi* extract induced a significant upregulation of ACE2 gene expression. For harmine, only the concentration of 3 μM resulted in an increase in ACE2 expression; however, this change did not reach statistical significance. Regarding TMPRSS2, the *B. caapi* extract did not alter its gene expression. However, harmine (10 µM) and harmaline (3 µM) significantly reduced TMPRSS2 expression.

## 4 Discussion

The literature has shown that SARS-CoV-2 is capable of infecting the central and peripheral nervous systems (Natoli et al., 2020; Brann et al., 2020; Paniz-Mondolfi et al., 2020). Although the CNS has the blood–brain barrier, SARS-CoV-2 is still able to cross it (Bale, 2015). A considerable number of studies have documented cases of long COVID patients experiencing attention deficit and impaired memory, language difficulties, and impaired visuospatial skills (Daroische et al., 2021; Del Brutto et al., 2021; Toniolo et al., 2021). These cognitive impairments were not limited to the acute phase of the disease or to severe cases (Vasile et al., 2023). In addition, potential factors contributing to the development of Alzheimer’s disease and related dementias include microglial inflammation (Oliveira et al., 2023), ischemic changes associated with COVID-19 (Solomon et al., 2020), and endothelial damage, which may impair the clearance of brain metabolites, including beta-amyloid peptides involved in Alzheimer’s disease (Jiang et al., 2024). In addition, degeneration and inflammation have been observed in the brains of individuals with COVID-19, including those without neurological symptoms. A correlation was found between marker genes for Alzheimer’s disease and genes that are upregulated during COVID-19 infection (Yang et al., 2021). Inflammatory biomarkers such as IL-6, IL-1, TNF-α, complement proteins, and galectin-3 have been proposed as shared prognostic indicators between SARS-CoV-2 infection and Alzheimer’s disease (Ciaccio et al., 2021; Rahman et al., 2021). It has been observed that the SARS-CoV-2 virus increases the production of cytokines, which can lead to the destruction of the myelin sheaths of nerve cells. This may trigger the synthesis of autoantibodies that attack neurons, causing damage to the brain’s anatomical structures and potentially contributing to the development of neurodegenerative diseases. Additionally, SARS-CoV-2 exacerbates inflammation, worsening the clinical condition in individuals already diagnosed with neurodegenerative diseases such as Alzheimer’s disease or multiple sclerosis. The secretion of pro-inflammatory cytokines can promote increased blood clot formation, leading to thrombosis, which may obstruct blood flow to the brain and cause an ischemic stroke (Kryńska et al., 2024). Studies have also described that COVID-19 induces an inflammatory response with high levels of IL-2 (Cantini et al., 2020), IL-4, IL-5 (Zhang et al., 2021), IL-6 (Cantini et al., 2020), IL-8, IL-10, IL-2R, TNF-α (Mulchandani et al., 2021), and IFN-γ (Zhang et al., 2021). We analyzed plasma samples from ICU and non-ICU patients hospitalized with a confirmed diagnosis of COVID-19, based on RT-qPCR and the presence of IgM/IgG antibodies against SARS-CoV-2. Consistent with its established role in the inflammatory response, IL-6 was detected in patient plasma and was significantly higher in ICU patients (Figure 1). These results provide contextual evidence of the clinical importance of IL-6 in severe COVID-19. However, these findings should not be interpreted as direct validation of our *in vitro* data; they only underscore the relevance of possible IL-6–mediated pathways in neuro-COVID.

The therapeutic potential of *Banisteriopsis caapi* extract, along with its main constituents, has been documented in the literature for conditions such as Parkinson’s disease and depression (Samoylenko et al., 2010), including its immunomodulatory and anti-inflammatory effects (da Silva et al., 2021; Santos et al., 2022). The β-carbolines, harmine, harmaline, and tetrahydroharmine, are main alkaloids found in *B. caapi* and exhibit antidepressant, antioxidant, and antigenotoxic activities, while inhibiting the isoforms of monoamine oxidases A and B (MAO-A and MAO-B), enzymes responsible for catecholamine degradation (Callaway et al., 1996; Yritia et al., 2002; McKenna and Riba, 2015). It has also been demonstrated that harmine, harmaline, and tetrahydroharmine promote neurogenesis in neurospheres prepared from progenitor cells harvested from the subventricular zone and dentate gyrus of adult mice (Morales-García et al., 2017). Moreover, all β-carbolines stimulated proliferation, migration, and differentiation of neural stem cells into adult neurons (Morales-García et al., 2017). In cultured neurons, they inhibited dual-specificity tyrosine-phosphorylation-regulated kinase 1A (DYRK1A), an enzyme implicated in the pathophysiology of various neurodegenerative diseases, and in the hippocampus of rats, they increased BDNF levels, a protein that plays an important role in neuroplasticity, neuronal survival, and differentiation (Fortunato et al., 2009; Frost et al., 2011). Anti-inflammatory effects of β-carbolines have also been described in the literature. It was demonstrated that harmine inhibited inducible NOS, COX-2, TNF-α, IL-6, IL-12, and other markers in LPS-induced BALB/c and C57BL/6 mouse macrophages (Jin et al., 2022). Uddin et al. (2020) designed, synthesized, and tested several harmaline analogs, observing that they inhibited purified human COX-2.

Considering our data both from volunteers and previous findings on β-carbolines and *B. caapi*, we initially decided to investigate whether exposure to *B. caapi* extract, harmine, or harmaline could lead to changes in pro-inflammatory cytokine levels or in the expression of SARS-CoV-2 receptors. For this study, we selected the SH-SY5Y cell line which has been used as a model for neuronal studies, including neuroinflammation, due to its various biochemical and functional neuronal properties (Joshi et al., 2006). First, we opted to evaluate whether the *B. caapi* extract or β-carbolines would be toxic to the SH-SY5Y cell culture. To do this, we incubated the cells with the compounds for 72 h and assessed cell viability using the MTT assay. We observed that the *B. caapi* extract was not toxic only at the 0.1% concentration (Figure 2A). In contrast, incubation with harmaline was toxic only at a concentration of 30 µM but did not alter cell viability at concentrations of 3 and 10 µM when compared to the control (Figure 2C), whereas harmine was not toxic at any of the concentrations tested (Figure 2B). Subsequently, we assessed cell viability (MTT assay too) following incubation with various concentrations of LPS at 48 h (Figure 2D). As expected, all studied concentrations of LPS were toxic to the cells. Therefore, we calculated the LPS IC50 (see Materials and Methods) and opted to use a dose of 130 μg/mL over a 48-h period.

Park et al. (2019) and Almohaimeed et al. (2024) induced neuroinflammation in the SH-SY5Y cell line using LPS. Park et al. (2019) observed that AS-6 (4-O-carboxymethylascoclorin) prevented LPS-induced neuroinflammation and cell death in co-cultured SH-SY5Y and BV2 cells by inhibiting the MAPK, NF-κB, and Akt pathways. Almohaimeed et al. (2024) evaluated IL-1β and IL-6 levels using ELISA in the SH-SY5Y cell line incubated with LPS and observed that treatment with taurine reduced the inflammatory cytokines. Regarding the effects of incubation with *B. caapi* extract and β-carbolines, we chose to maintain the induction model with LPS for 48 h, and *B. caapi* extract or β-carbolines were added to the culture medium 24 h before LPS, totaling 72 h of *B. caapi* extract or β-carboline incubation. We also observed a significant increase (p < 0.05) in NF-κB expression and in IL-6 and TNF-α levels, as evaluated by RT-qPCR and ELISA, respectively, after 48 h of LPS incubation (Figures 3, 4). Therefore, the neuroinflammation model induced by LPS incubation in 2D culture of SH-SY5Y cells is well established in the literature. Concerning the inflammatory mediator NF-κB, our RT-qPCR analysis revealed that *B. caapi* extract and harmine significantly decrease the LPS-induced transcription of NF-κB in SH-SY5Y cells, indicating modulation of the NF-κB axis upstream of cytokine release. It has been demonstrated that harmine suppresses NF-κB activation in RAW 264.7 macrophages by inhibiting p65 phosphorylation and nuclear translocation following TNF-α or LPS stimulation, resulting in reduced mRNA and protein levels of TNF-α, IL-1β, and IL-6 (Xin et al., 2017). Zhai et al. (2025) also reported that harmine inhibits the expression and secretion of LPS-induced inflammatory cytokines (IL-6, IL-1β, and TNF-α) and reduces inflammatory cell infiltration in mouse lung tissue. Moreover, the authors demonstrated that harmine attenuates inflammation by suppressing *CSF3* transcription and expression, ultimately inhibiting activation of the MAPK/NF-κB signaling pathway in LPS-stimulated RAW 264.7 cells. NF-κB is a key upstream regulator of inflammatory cytokine production and, upon LPS stimulation, rapidly induces primary response genes such as IL-6 and TNF-α, a process dependent on chromatin accessibility and histone modifications that facilitate its binding to target promoters (Medzhitov and Horng, 2009). Therefore, reducing NF-κB activity may be a decisive factor in attenuating the inflammatory response, and additional analyses will be valuable for future investigations. We also observed that harmine reduced basal and LPS-induced levels of IL-6 and TNF-α. Harmaline (3 μM) also reduced the basal and LPS-induced levels of IL-6 and TNF-α. However, doses of 10 µM caused a reduction only in the basal levels of both cytokines. In contrast, the *B. caapi* extract, at a concentration of 0.1%, reduced only TNF-α induced by LPS, but there was no difference in the basal or induced level of IL-6, after extract incubation (Figure 4). A recent study showed that β-carbolines found in the *B. caapi* extract had a cytotoxic effect at high concentrations but also exerted an important anti-inflammatory effect at low levels in microglial BV-2 cells by decreasing pro-inflammatory cytokine release (Santos et al., 2022). In the same study, harmaline significantly decreased the release of all pro-inflammatory cytokines, except for IL-6. Treatment with tetrahydroharmine notably reduced the production of IL-6, TNF-α, and IFN-γ at low concentrations. In contrast, harmine had little effects on most cytokines, except for a gradual significant reduction in TNF-α release at the highest doses (Santos et al., 2022). The authors did not observe changes in the cytokine profile after incubation with the *B. caapi* extract. We believe that the differences observed in the inhibition profile of cytokine levels may be related to the concentration of beta-carbolines or extract, cell culture, and incubation time. For example, in the aforementioned study, the authors incubated Bv-2 cells (microglial line) with β-carbolines or *B. caapi* extract for 2 h. Our study used the SH-SY5Y cell line (human neuroblastoma), and the incubation of *B. caapi* extract and beta-carbolines was 72 h in total. In addition, we used an LPS-induced inflammation model. Despite the differences, both studies showed a reduction in the levels of inflammatory cytokines after incubation with β-carbolines.

Subsequently, we evaluated whether the *B. caapi* extract or harmine and harmaline could alter the expression of SARS-CoV-2 receptors by RT-qPCR (Figure 5). SARS-CoV-2 can enter the cell through various mechanisms such as ACE2 (Hörnich et al., 2021), transmembrane serine protease II (TMPRSS2) (Fraser et al., 2022), and TMPRSS11D or the cellular protease furin (Glowacka et al., 2011; Hoffmann et al., 2020; Kishimoto et al., 2021). In this context, ACE2 upregulation observed after treatment with the *B. caapi* extract in SH-SY5Y cells does not necessarily imply a deleterious effect in the setting of SARS-CoV-2. Beyond functioning as a viral entry receptor, ACE2 is a key negative regulator of the renin–angiotensin system, converting Ang II into Ang-(1–7) and activating the ACE2/Ang-(1–7)/Mas axis, which exerts vasodilatory, anti-inflammatory, antioxidant, and antifibrotic actions in several organs, including the lung, cardiovascular system, and brain (Li et al., 2015). Experimental models of acute lung injury show that ACE2 deficiency aggravates inflammation and tissue damage, whereas ACE2 overexpression or treatment with recombinant ACE2 attenuates injury and improves outcomes, and viral binding to ACE2 is thought to worsen disease by promoting ACE2 internalization and downregulation, thereby shifting the balance toward the deleterious ACE/Ang II/AT1R axis (Ravindra et al., 2020). In the central nervous system, ACE2 and the Ang-(1–7)/Mas axis have been implicated in neuroprotection, modulation of neuroinflammation, and preservation of cognitive function, largely by counteracting Ang II–mediated oxidative stress, glial activation, and pro-inflammatory signaling (Bennion et al., 2015). In this scenario, a moderate increase in ACE2 expression in non-infected neuronal-like cells—particularly in the absence of parallel induction of key entry cofactors—may be interpreted as a neutral or even potentially beneficial modulation of host-protective pathways rather than as an intrinsically pro-viral effect. In line with this interpretation, our data show that *B. caapi* extract and harmine at 3 µM exerted a more modest effect, increasing ACE2 expression in SH-SY5Y cells without significantly modulating TMPRSS11D or furin, and under conditions in which TMPRSS2 expression was minimal or undetectable. These findings suggest that β-carboline–containing preparations may preferentially engage the protective ACE2/Ang-(1–7)/Mas pathway in neuronal-like cells while not clearly enhancing the full complement of factors required for efficient SARS-CoV-2 entry.

Interestingly, we also observed that harmine and harmaline reduced basal TMPRSS2 expression in SH-SY5Y cells (Figure 5D). To the best of our knowledge, there are no previous experimental studies directly evaluating the impact of β-carbolines on TMPRSS2 transcription. However, TMPRSS2 is an androgen-responsive and inflammation-sensitive protease whose expression can be modulated by sex steroids and by bacterial products such as LPS, flagellin, and Pam3Cys in airway epithelial cells (Deng et al., 2021; Treppiedi et al., 2022; Ruffin et al., 2021; Schwerdtner et al., 2023). In addition, high-throughput and network-based screens have identified several small molecules that downregulate TMPRSS2 expression and thereby limit SARS-CoV-2 entry, supporting the concept that host-directed modulation of this protease is pharmacologically feasible (Chen et al., 2021). Natural products have emerged as candidates in this context, and harmine, in particular, exhibits potent anti-inflammatory and signaling-modulatory properties, including inhibition of NF-κB, MAPK, and STAT1 pathways and attenuation of TLR-driven inflammatory responses *in vitro* and *in vivo* (Jin et al., 2022; Ruan et al., 2022; Zhai et al., 2025). On this basis, it is plausible that the decrease in TMPRSS2 expression elicited by harmine and harmaline in SH-SY5Y cells reflects indirect effects on intracellular signaling cascades that normally promote TMPRSS2 expression, suggesting that β-carboline–containing preparations may potentially restrict SARS-CoV-2 neurotropism by simultaneously modulating ACE2 and downregulating a key spike-activating protease.

Although these findings are promising, when considered collectively, they highlight the need for further investigation to elucidate the potential antiviral activity of β-carbolines or *B. caapi* extract, considering both *in vitro* and *in vivo* models, along with the specific virus type or strain. Together, these findings highlight the importance of investigating both whole-plant extracts, considering the potential interactions among their constituents, and isolated compounds individually as each may contribute differently to the overall biological activity. Accordingly, further studies are needed to elucidate the potential antiviral effects of β-carbolines and *B. caapi* extract, considering both *in vitro* and *in vivo* approaches, along with the specific viral species or strain under investigation.

## 5 Conclusion

β-Carbolines reduced both basal and LPS-induced IL-6 and TNF-α production in SH-SY5Y cells, supporting their potential neuroprotective activity under neuroinflammatory conditions. Harmine also significantly decreased NF-κB transcription, which is a key upstream regulator of cytokine expression. In contrast, the *B. caapi* extract selectively attenuated LPS-induced TNF-α release and similarly downregulated NF-κB transcription. Harmine, harmaline, and *B. caapi* extract did not significantly alter TMPRSS11D or furin, and harmaline showed no effect on ACE2. Notably, the extract upregulated ACE2, while harmine produced only a modest, non-significant increase. Harmine and harmaline also reduced TMPRSS2 expression in SH-SY5Y cells. These findings indicate that β-carbolines and *B. caapi* extract may be promising for reducing neuroinflammation *in vitro*. The inclusion of IL-6 results from COVID-19 patients serves to highlight the clinical relevance of this cytokine in disease severity but should not be interpreted as direct validation of the *in vitro* results. Together, these observations support further investigation into the potential role of these compounds in the context of neuro-COVID.

## Data Availability

The raw data supporting the conclusions of this article will be made available by the authors, without undue reservation.
